# Inhalational Anthrax Outbreak among Postal Workers, Washington, D.C., 2001

**DOI:** 10.3201/eid0810.020330

**Published:** 2002-10

**Authors:** Puneet K. Dewan, Alicia M. Fry, Kayla Laserson, Bruce C. Tierney, Conrad P. Quinn, James A. Hayslett, Laura N. Broyles, Andi Shane, Kevin L. Winthrop, Ivan Walks, Larry Siegel, Thomas Hales, Vera A. Semenova, Sandra Romero-Steiner, Cheryl Elie, Rima Khabbaz, Ali S. Khan, Rana A. Hajjeh, Anne Schuchat, members of the Washington, D.C., Anthrax Response Team

**Affiliations:** * Centers for Disease Control and Prevention, Atlanta, Georgia, USA; † Washington, D.C. Department of Health, Washington, D.C., USA

**Keywords:** bioterrorism, *Bacillus anthracis*, postal facility, inhalational anthrax

## Abstract

In October 2001, four cases of inhalational anthrax occurred in workers in a Washington, D.C., mail facility that processed envelopes containing *Bacillus anthracis* spores. We reviewed the envelopes’ paths and obtained exposure histories and nasal swab cultures from postal workers. Environmental sampling was performed*.* A sample of employees was assessed for antibody concentrations to *B. anthracis* protective antigen. Case-patients worked on nonoverlapping shifts throughout the facility. Environmental sampling showed diffuse contamination of the facility, suggesting multiple aerosolization events. Potential workplace exposures were similar for the case-patients and the sample of workers. All nasal swab cultures and serum antibody tests were negative. Available tools could not identify subgroups of employees at higher risk for exposure or disease. Prophylaxis was necessary for all employees. To protect postal workers against bioterrorism, measures to reduce the risk of occupational exposure are necessary.

In October 2001, four cases of inhalational anthrax occurred in employees at the Washington, D.C., Postal Processing and Distribution Center (DCPDC) (1,2). These cases were part of a multistate outbreak of inhalational and cutaneous anthrax associated with intentional distribution of envelopes containing *Bacillus anthracis* spores to media and federal government offices (2–4). Together, these represent the first reported cases of inhalational anthrax in postal workers and the first reported outbreak of inhalational anthrax caused by occupational exposure in the United States since 1957 (5,6).

The investigation and public health response to this outbreak of inhalational anthrax are reported here. The urgent public health response was directed at preventing new cases of inhalational anthrax through the use of prophylactic antimicrobial drugs for persons potentially exposed to *B. anthracis* spores. The public health response also provided useful information about occupational exposure to aerosolized spores in this type of workplace and the performance of potential tools for determining exposure, such as work history, nasal swabs, immune response markers, and environmental sampling.

## Methods

### Setting and Background

On October 15, 2001, in an office of the Washington, D.C., U. S. Capitol complex, an envelope addressed to Senator Tom Daschle, intentionally contaminated with *B. anthracis* spores*,* was opened. This event occurred 2 weeks after a report from Florida of the first-ever inhalational anthrax cases related to envelopes containing *B. anthracis* spores; those cases occurred in employees of a media company (3). The Washington, D.C., Department of Health (DCDOH), Office of the Attending Physician, U.S. Capitol, and the Centers for Disease Control and Prevention (CDC) immediately initiated a multiagency public health response and epidemiologic investigation (7). Enhanced surveillance activities for inhalational anthrax in the national Capitol area were established through a cooperative effort of the DCDOH, Virginia Department of Health, Maryland Department of Health and Mental Hygiene, and CDC.

The epidemiologic investigation determined that the *B. anthracis*–contaminated envelope addressed to Sen. Daschle was processed on October 12 at the DCPDC before entering the Capitol mail distribution system. Late on October 19, a DCPDC employee was admitted to a Virginia hospital with a diagnosis of suspected inhalational anthrax. The CDC team visited the DCPDC on October 20. The suspected case-patient worked in an area of the DCPDC where the envelope had not been processed; he also worked in a second mail facility in Maryland. The diagnosis of inhalational anthrax was confirmed on October 21 (1,2). The DCPDC and the second mail facility in Maryland were closed on October 21. On October 20–22, three additional cases of suspected inhalational anthrax were identified in the DCPDC employees; two of these patients died (Table 1). *B. anthracis* grew from blood cultures from all patients within 24 hours. *B. anthracis* was confirmed by *B. anthracis*–specific polymerase chain reaction assay at CDC (2).

**Table 1:** Characteristics of inhalational anthrax cases among employees of the Washington, D.C., Processing and Distribution Center^a^

	Case 1	Case 2	Case 3	Case 4
Age (yrs)	56	56	55	47
Race	AA	AA	AA	AA
Date symptoms began	10/16	10/16	10/16	10/16
Date of suspected IA diagnosis	10/19	10/20	10/21	10/22
Date IA confirmed	10/21	10/22	10/23	10/26
Underlying medical conditions	No	No	Yes^b^	Yes^c^
Death due to IA (date)	No	No	Yes (10/21)	Yes (10/22)

^a^AA = African-American, IA=inhalational anthrax. ^b^Diabetes mellitus, sarcoidosis. ^c^Asthma.

A second envelope with *B. anthracis* spores, addressed to Sen. Patrick Leahy, was identified on November 16. This envelope was recovered from a sealed drum containing U.S. Capitol mail quarantined on October 17, 2001.

### Postexposure Prophylaxis

 After confirmation of the first case of inhalational anthrax in a DCPDC employee, antimicrobial postexposure prophylaxis (PEP) was recommended to all DCPDC employees and visitors to the nonpublic mail-processing area (3,8). DCPDC employees who had been absent from work >24 hours in the past 7 days were contacted to identify any additional cases and inform workers of the recommendation for PEP. Beginning October 21, workers were given a 10-day supply of antimicrobial therapy, pending further investigation. DCPDC employees returned to the public health department antimicrobial agent distribution centers to receive an additional 50-day supply of antimicrobial therapy. All DCPDC employees were offered free medication from the U.S. National Pharmaceutical Stockpile at D.C. General Hospital through a cooperative effort of the DCDOH and the U.S. Public Health Service. Employees could choose to obtain appropriate medication from other sources. The United States Postal Service (USPS) notified employees from the DCPDC about the recommendation for postexposure prophylaxis and urged them to comply. Information on the symptoms of inhalational anthrax, the biology of *B. anthracis*, and possible adverse effects from antimicrobial agents was distributed to postal workers. The number of employees who obtained antimicrobial therapy from D.C. General Hospital, the Virginia Department of Health, and Maryland Department of Health and Mental Hygiene was recorded.

### Postal System Assessment

 In collaboration with the USPS and the Postal Inspection Service, we assessed routine mail-handling procedures and reviewed the path of the two envelopes that were known to contain *B. anthracis* spores. From unique envelope markings, postal inspectors determined the time of automated envelope processing and the machinery used during processing. To establish the number of employees potentially exposed during the passage and processing of the two envelopes, DCPDC employee work zone locations, job descriptions, and assigned work shifts were obtained from USPS administrative data.

### Case Exposure Histories

 We interviewed surviving case-patients and close associates of those who died by using a standard exposure questionnaire. Case-patients were assessed for job description, work and break locations, travel and medical history, and potential exposure to natural reservoirs of *B. anthracis* spores. Timecard logs established exact times of work during October 11–21.

### Environmental Assessment

 Beginning October 23, the DCPDC facility was sampled extensively with a combination of surface wipes, surface vacuum samples, and air vacuum samples, reported in detail elsewhere (9–11).

### Nasal Swab Cultures

Nasal swab cultures from the DCPDC employees and those who reported visiting that facility during the period October 10–21 were obtained on October 21–22 during the distribution of antimicrobial therapy. Specimens were processed by standard microbiologic methods at the Maryland Department of Health laboratory (12).

### Serologic and Exposure Survey

We conducted a survey to evaluate occupational exposures of workers and determine whether there was evidence of immunologic response to *B. anthracis* protective antigen. Exposure histories and serum samples were obtained from a convenience sample of DCPDC employees who went to D.C General Hospital on October 29–30 for their additional 50-day supply of antimicrobial therapy. Each participant was asked to allow a serum sample to be collected and to be individually interviewed with the standardized exposure questionnaire used for case-patients. Informed consent was obtained from all participants.

One blood sample was obtained from each participant. The serum was separated and stored at 4°C. Anti-protective antigen immunoglobulin G (anti-PA IgG) antibody concentrations in serum specimens were determined by a quantitative enzyme-linked immunoassay described in detail elsewhere (13).

### Comparison of Case-Patients and Survey Participants

 We compared exposure histories and underlying diseases of the case-patients with the sample of surveyed workers to clarify factors that may have contributed to the four cases of inhalational anthrax at DCPDC. Data from the standardized exposure questionnaire from the DCPDC cases and the other sampled employees were compared by a case-control analysis with two-tailed Fisher exact tests for dichotomous variables or the Wilcoxon signed-ranks test for continuous variables; p values of <0.05 were considered significant.

## Results

### Postexposure Prophylaxis

 Of 2,403 employees at the DCPDC, 1,870 (78%) were recorded as receiving a 50-day supply of antimicrobial therapy at DCDOH, Virginia Department of Health, or Maryland Department of Health and Mental Hygiene postexposure prophylaxis distribution centers. Five members of the CDC team received PEP.

### Postal System Assessment

**Figure 1:**
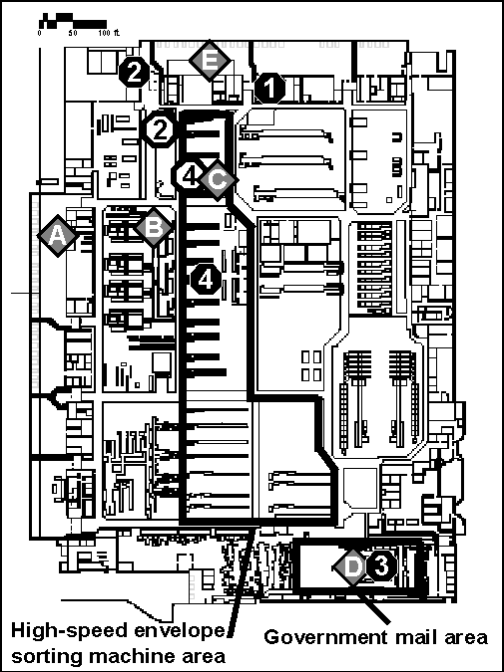
Floor map of the Washington, D.C., Postal Processing and Distribution Center with the known locations of the two *B. anthracis–*contaminated envelopes (gray diamonds with letters) and work locations of case-patients (black circles with numbers) in the facility on October 12, 2001. The estimated location of case-patients during the time of processing the contaminated envelopes at point C, when the letters were processed by the high-speed sorter machine, are shown as open circles. The main processing area of the facility, containing all of the high-speed sorter machines, and the government mail section of the facility are marked.

The DCPDC is a 500,000-square foot facility (Figure 1). Approximately 59 million pieces of incoming mail were processed at the DCPDC during October 11–21. The two contaminated envelopes entered the DCPDC on the evening of October 11 or early morning of October 12 in a tray of envelopes originating at the processing and distribution center in Trenton, New Jersey. This tray was taken from the dock (Figure 1, point A) to a large tray-sorting machine (Figure 1, point B) and then moved to a high-speed envelope-sorting machine known as a delivery bar-code sorter (DBCS). The DBCS moves up to 30,000 letters per hour into a series of bins for subsequent distribution. At the end of each work shift, the DBCS is cleaned by a procedure that blows compressed air (70 lbs per square in) into the machine. Unique processing markings on the two envelopes showed that DBCS number 17 (Figure 1, point C) sorted both envelopes on October 12 between 7:05 and 7:30 a.m. The two letters appeared to be processed within minutes of each other.

The envelope addressed to Sen. Daschle was sorted into a bin destined for the U.S. Capitol, taken out of the DBCS number 17, and moved to the government mail section (Figure 1, point D). The government mail section handles distribution of all letters to U.S. government addresses in the metropolitan D.C. area. Routinely, in a process known as riffling, envelopes are flipped through individually for manual confirmation of appropriate sorting. The envelope addressed to Sen. Daschle transited the government mail section on October 12 between 7:30 a.m. and noon, at which time it was dispatched from the loading dock (Figure 1, point E) to the U.S. Capitol’s mail distribution facility.

The envelope addressed to Sen. Leahy was incorrectly sorted as destined for the U.S. State Department, State Annex 32, which has an independent small mail-processing facility in Virginia. The exact path of this envelope is unclear from October 12 to 17. On October 17, the Federal Bureau of Investigation quarantined all remaining U.S. Capitol mail and placed it into sealed drums for further investigation; the Leahy envelope was found in one of these drums on November 16. The letter appeared to be leaking. Routine procedures for redirecting incorrectly sorted envelopes destined for a U.S. government address usually involve employees in the DCPDC government mail section. If routine procedures had been followed, and the envelope were recognized as incorrectly sorted, the envelope would have been manually redirected in the DCPDC government mail section in the period October 12–16.

A fifth case of suspected inhalational anthrax in a postal worker in Virginia was reported on October 25, 2001. The case-patient worked in State Annex 32 (3). Whether the envelope to Sen. Leahy remained in the DCPDC or transited through the State Annex 32 is not known. Mail destined for the State Annex 32 was sorted at the same time in DBCS number 17 as the envelope addressed to Sen. Leahy. Environmental sampling results from State Annex 32 showed widespread contamination with *B. anthracis* spores, similar to the DCPDC.

Of the 1,961 employees of the DCPDC nonpublic mailroom area, 610 (31%) were assigned to work in one of the same work zones as the four case-patients from this facility. During the time of the two envelopes’ passage and processing through the DCPDC, approximately 108 (6%) worked in the main processing area with the DBCS machines between 12 a.m.–12 p.m. on October 12, and 87 (4%) employees worked in the government mail section (Figure 1). Two of the four case-patients worked in one of these work zones. The attack rate for inhalational anthrax in these combined areas was 1% (2/195).

### Exposure Histories

The clinical characteristics of the case-patients have been described.(2) All four case-patients from the DCPDC were African-American; case-patient 5 from the mail-processing facility for the State Department was white (Table 1). Only two case-patients had underlying medical conditions. Case-patient 3 had adult-onset diabetes mellitus and a 30-year history of sarcoidosis, although the patient was not on medication for either condition (14). Case-patient 4 had a diagnosis of asthma and was periodically treated with bronchodilators.

**Figure 2:**
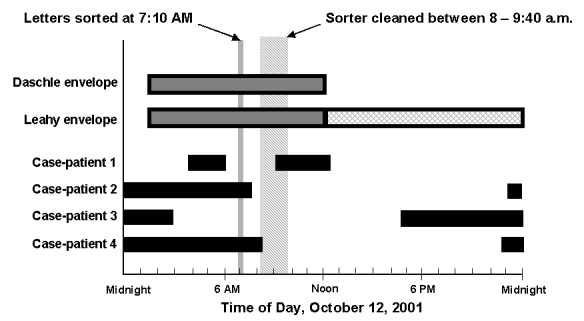
Comparing the time period that the case-patients were at the Washington, D.C., Postal Processing and Distribution Center (solid black bars) to the time period that the two envelopes containing *B. anthracis* spores were processed at the facility (gray bars = known location, gray hatched bars = unknown location) on October 12, 2001. The time that the high-speed sorting machine (delivery bar-code sort number 17) was cleaned, by blowing compressed air into the machine, is denoted by the gray striped area.

Two of the four case-patients from DCPDC worked within several meters of the path of the processed envelopes (Figures 1 and 2). Only one case-patient routinely worked directly with high-speed envelope-sorting machinery, including routine overtime on DBCS number 17. At the time the two contaminated envelopes were sorted in the DCPDC by DBCS number 17, only case-patients 2 and 4 were physically in the DCPDC facility (Figure 2). However, case-patient 1 returned during the window of time when DBCS number 17 was cleaned by blowing compressed air into the machine, between 8:00 a.m. and 9:40 a.m. Case-patient 3 returned to work in the government mail section (Figure 1, point D) at 8:00 p.m. on October 12.

### Environmental Sampling

Diffuse environmental contamination with *B. anthracis* was found throughout the nonpublic mail-processing area of the DCPDC, particularly on DBCS number 17 and in the government mail section of the facility (9–11). In addition, two supply air ventilation diffusers, located above the area where two of the case-patients worked, were contaminated with *B. anthracis* spores (10,11). None of the samples taken from the public area of the facility were positive for *B. anthracis* spores.

### Nasal Swab Cultures

 Nasal swab cultures from 3,110 DCPDC employees and visitors, collected 9–10 days after the two envelopes were processed at the DCPDC, were negative for growth of *B. anthracis*.

### Seroprevalence and Exposure Survey

 On October 29–30, a total of 1,657 employees and visitors to the DCPDC went to D.C. General Hospital to receive additional antibiotic supplies. Of these, 784 (47%) were asked to participate in the survey; 224 (29%) of 784 DCPDC employees participated. Serum samples were obtained from 202 (94%). None of the 202 serum samples had significant detectable specific IgG antibody concentrations of anti-PA IgG, including the three participants who reported a remote history of anthrax vaccination.

 The routine work activities of case-patients were also relatively common for the surveyed DCPDC workers (Table 2). Fifty-four percent reported that they manually sorted mail, and 39% reported that they riffled mail. Seven percent of survey participants reported that they riffled mail on October 12. Few employees reported the use of masks (7%), although 47% of survey participants and 50% of case-patients reported using gloves.

**Table 2:** Comparison of characteristics and potential exposures among case-patients and survey participants from the Washington, D.C., Processing and Distribution Center^a^

Characteristics and potential exposures	Cases (n=4) N (%)	Participants (n=214) N (%)	OR (95% CI)	p value
Characteristics				
Median years of age (range)	56 (47–56)	49 (25–71)		0.31
Underlying medical conditions				
Any underlying condition	2 (50)	51 (24)	3.2 (0.44 to 23.3)	0.23
Chronic lung disease^b^	1 (25)	20 (9)	3.6 (0.38 to 38.8)	0.25
Diabetes mellitus	1 (25)	18 (8)	3.6 (0.38 to 38.8)	0.25
Heart disease	0	15 (7)		0.58
Liver disease	0	4 (2)		0.78
Recent corticosteroid use^c^	0	9 (4)		0.78
Potential exposures				
Manually sorted mail	3 (75)	115 (54)	2.6 (0.23 to 64.8)	0.63
Riffled mail	3 (75)	84 (39)	4.6 (0.4 to 116.9)	0.30
Worked on sorter machine	1 (25)	75 (35)	0.61 (0.02 to 6.8)	1.00
*B. anthracis* vaccination	0	3 (1)		1.00
Worked on 10/12/2001	4 (100)	178 (83)		0.48

^a^OR, odds ratio. CI, confidence interval. ^b^Chronic lung disease includes asthma, bronchitis, emphysema, and obstructive lung disease. ^c^Use within previous 2 weeks.

### Comparison of Case-Patients and Survey Participants

 Differences in underlying medical conditions or workplace exposures between the DCPDC case-patients and the survey participants were not statistically significant (Table 2). With sarcoidosis included as a chronic lung disease, more case-patients had chronic lung disease than did survey participants (50% vs. 9%; odds ratio 9.65; 95% confidence interval 1.29 to 72.2; p=0.01). None of the case-patients currently smoked cigarettes, compared with 24% of the participants. Specific mail-handling activities such as manually sorting mail or working on a sorting machine also did not differ. No case-patients and few (10%) of the serosurvey participants handled bulk mail.

## Discussion

At least two letters containing *B. anthracis* spores were processed at the DCPDC facility on October 12, 2001, resulting in an outbreak of four cases of inhalational anthrax in postal employees who worked in that facility. Our investigation demonstrated widespread contamination of the facility with *B. anthracis* spores, including areas through which the two letters were unlikely to have traveled. The case-patients did not all work directly along the path of the contaminated envelopes as they were processed through the facility on October 12, and two patients were not even in the building at the time of mechanical sorting. Therefore, inhalational anthrax likely resulted from multiple aerosolization events, including processing of the letters through the high-speed sorting machine, manual sorting and riffling of mail, and cleaning the high-speed sorting machine by blowing compressed air into it. Evaluation of re-aerosolization of *B. anthracis* spores at the DCPDC, conducted after partial cleaning of the high-speed sorter that processed the *B. anthracis–*containing envelopes, DBCS number 17, identified ongoing low-level aerosolization after the machine was turned on, suggesting aerosolized spores were likely present at some level throughout the 10 days from October 12 until the facility closed on October 21 (15).

Before recognition of inhalational anthrax among postal workers in Washington, D.C., and New Jersey, two cases of inhalational anthrax and several cases of cutaneous anthrax were identified in Florida and New York in employees of media companies; the latter cases were associated with contaminated envelopes postmarked at the Trenton Processing and Distribution Center (PDC) September 18, 2001 (2,7). Despite this, the first recognition of inhalational disease in the postal service occurred in Washington, D.C., associated with letters processed in the Trenton PDC, October 9, 2001. Why the envelopes processed in October resulted in cases of inhalational anthrax among postal workers while those processed in September did not is unclear. A likely possibility is that the characteristics of the *B. anthracis* preparation or the condition of the envelope(s) at the time of transits through the DCPDC in October (or both) differed from that in September (16). The events that occurred in October in Washington, D.C., suggest the need to ensure that future bioterrorism events involving *B. anthracis* contamination of envelopes incorporate new understanding of the aerosolization potential in the PDC environment, the need for extensive traceback of contaminated envelopes, and broad initiation of prophylaxis to all persons potentially exposed to spores.

Given the widespread contamination of the DCPDC and the likelihood of multiple aerosolization events, why the four case-patients developed inhalational anthrax but other workers in the same facility did not is not clear. Some underlying medical conditions may make persons more susceptible to inhalational anthrax during the initial period after exposure, although we were unable to demonstrate this conclusively in this investigation, primarily because of small numbers. Many employees in the DCPDC performed activities at work that might have resulted in aerosolization of spores. Given the potential for a long incubation period, especially after low-dose exposures (17,18), and documented re-aerosolization (15), many additional cases of inhalational anthrax were likely prevented by the postexposure prophylaxis given to all facility employees 9 days after the two envelopes were processed at the DCPDC.

More than 2,000 postal employees were advised to take 60 days of antimicrobial agents to prevent inhalational anthrax. We used currently available methods, including nasal swab cultures, a serologic assay, and environmental sampling, to identify DCPDC workers who were exposed to *B. anthracis* spores. While the environmental sampling and exposure survey suggested that many persons could have been exposed to *B. anthracis*, neither the nasal swab cultures nor serologic survey could reliably identify subgroups of DCPDC workers who were exposed and thus at higher risk of developing inhalational anthrax. Therefore, among DCPDC employees, postexposure prophylaxis was necessary for all workers in the facility. Until better methods to determine exposure to *B. anthracis* and to assess risk factors for development of inhalational anthrax are available, broad implementation of to all persons potentially exposed will be necessary. Vaccines may play a role in postexposure prophylaxis, in addition to their recognized role in preexposure prophylaxis for persons from selected high-risk occupations.

 Serologic analyses for *B. anthracis* have been developed to confirm seroconversion after anthrax vaccine administration (19) but have been used to provide retrospective confirmation of cutaneous *B. anthracis* infection (20,21). During this bioterrorism event, the anti-PA IgG antibody assay was developed, validated, and used to confirm clinical cases of disease for the first time; the assay had good sensitivity and specificity to detect clinical disease (13). As demonstrated here, this IgG assay was not able to determine whether persons without clinical disease were exposed or infected with *B. anthracis*; whether an anti-PA IgM antibody assay would improve sensitivity is unknown. Although we did not obtain serum specimens from DCPDC employees at a longer interval after exposure, an investigation of employees on Capitol Hill failed to detect anti-PA IgG antibody as late as 4 weeks after exposure (7). Additionally, all DCPDC participants in the survey had been taking antimicrobial agents since October 21–22; the antimicrobial agents may have blunted the immune response. Nasal swab cultures collected 9 days after the two envelopes were processed at the DCPDC were also negative. These findings may be due to many factors, including low exposure to spores, the transient nature of *B. anthracis* spores in the nasal passages, or the low sensitivity of this assay. Previous studies have isolated *B. anthracis* in nasal passages long periods after exposure (4,6); however, the characteristics of the spores disseminated throughout the DCPDC may not be similar to those previously studied. Environmental sampling detected *B. anthracis* spores in the DCPDC but at this time cannot determine the inoculum size. In addition, the correlation between environmental culture data and risk for disease remains unclear. In light of these limitations, multiple criteria, including epidemiologic and environmental results, should be considered when deciding whether prolonged postexposure prophylaxis is warranted.

 Because of the unprecedented nature of this outbreak, the risks of inhalational anthrax associated with exposure to *B. anthracis* spores were unknown when we began our investigation. We have learned that the preparations of *B. anthracis* spores used in this event had a high potential for diffuse aerosolization, especially in settings such as the DCPDC. Our current diagnostic tools are limited in their ability to identify persons who were exposed to spores and likely to become ill; future studies are needed to improve these tools. In spite of this, many procedures that increased the likelihood of spore dissemination in PDC facilities have been identified and can be modified to reduce the risk to workers in the event of a future event. For example, the practice of blowing compressed air into sorting machines could be discontinued, and use of appropriate respiratory protective equipment could be encouraged (22). Occupational safety of postal workers from bioterrorism and other health hazards can be enhanced with attention to engineering, procedural safety measures, and personal protective equipment. The public health response to future bioterrorism events that involve *B. anthracis* spores should include extensive traceback of contaminated envelopes and broad use of prophylactic measures to prevent disease.

## References

[1] Centers for Disease Control and Prevention. Update: investigation of bioterrorism-related anthrax and interim guidelines for exposure management and antimicrobial therapy, October 2001. MMWR Morb Mortal Wkly Rep. 2001;50:909–19. 11699843

[2] Jernigan JA, Stephens DS, Ashford DA, Omenaca C, Topiel MS, Galbraith M, Bioterrorism-related inhalational anthrax: the first 10 cases reported in the United States. Emerg Infect Dis. 2001;7:933–44. 11747719 10.3201/eid0706.010604 PMC2631903

[3] Centers for Disease Control and Prevention. Update: investigation of bioterrorism-related anthrax and interim guidelines for clinical evaluation of persons with possible anthrax. MMWR Morb Mortal Wkly Rep. 2001;50:941–8. 11708591

[4] Centers for Disease Control and Prevention. Update: investigation of anthrax associated with intentional exposure and interim public health guidelines, October 2001. MMWR Morb Mortal Wkly Rep. 2001;50:889–93. 11686472

[5] Plotkin SA, Brachman PS, Utell M, Bumford FH, Atchison MM. An epidemic of inhalation anthrax, the first in the twentieth century: I. Clinical features. 1960. Am J Med. 2002;112:4–12. 10.1016/S0002-9343(01)01050-6 11812400 PMC7172370

[6] Brachman PS, Plotkin SA, Bumford FH, Atchison MM. An epidemic of inhalation anthrax: the first in the twentieth century. II. Epidemiology. Am J Hyg. 1960;72:6–23. 13803672 10.1093/oxfordjournals.aje.a120135

[7] Hsu PV, Lukacs SL, Handzel T, Hayslett J, Harper S, Hales T, Opening a *Bacillus anthracis–*Containing Envelope, Capitol Hill, Washington, D.C.: The Public Health Response. Emerg Infect Dis. 2002;8:1039–43. 12396912 10.3201/eid0810.020332 PMC2730304

[8] Bell DM, Kozarsky PE, Stephens DS. Clinical issues in the prophylaxis, diagnosis, and treatment of anthrax. Emerg Infect Dis. 2002;8:222–5. 11897081 10.3201/eid0802.01-0521 PMC2732453

[9] Centers for Disease Control and Prevention. Evaluation of *Bacillus anthracis* contamination inside the Brentwood Mail Processing and Distribution Center—District of Columbia, October 2001. MMWR Morb Mortal Wkly Rep. 2001;50:1129–33. 11824387

[10] Sanderson W, Stoddard R, Echt A, McCleery RE, Picitelli CA, Kim D, Evaluation of *Bacillus anthracis* contamination inside the Brentwood Post Office, Washington, D.C. Report to U.S. Postal Service. Cincinnati: National Institute for Occupational Safety and Health; 2001.

[11] Sanderson W, Hein M, Taylor L, Curwin B, Kinnes G, Hales T, Second evaluation of *Bacillus anthracis* contamination inside the Brentwood Mail Processing and Distribution Center, District of Columbia. Report to the U.S. Postal Service. Cincinnati: National Institute for Occupational Safety and Health; 2002.

[12] Murray PR. Manual of clinical microbiology. 7th edition. Washington: ASM Press; 1999.

[13] Quinn CP, Semenova VA, Ellie EM, Romero-Steiner S, Greene C, Li H, A specific, sensitive, and quantitative enzyme-linked immunosorbent assay for human immunoglobulin G antibodies to anthrax toxin protective antigen. Emerg Infect Dis. 2002;8:1103–10. 12396924 10.3201/eid0810.020380 PMC2730307

[14] Brachman PS, Pagana JS, Albrink WS. Two cases of fatal inhalation anthrax, one associated with sarcoidosis. N Engl J Med. 1961;265:203–8.

[15] Dull P, Wilson K, Kournikakis W, Boulet C, Ho J, Ogston J, *Bacillus anthracis* Aerosolization Associated with a Contaminated Mail Sorting Machine. Emerg Infect Dis. 2002;8:1044–7. 12396913 10.3201/eid0810.020356 PMC2730297

[16] Broad WJ, Johnston D. Anthrax sent through mail gained potency by the letter. New York Times 2002 May 7; Sect. A:1.

[17] Meselson M, Guillemin J, Hugh-Jones M, Langmuir A, Popova I, Shelokov A, The Sverdlovsk anthrax outbreak of 1979. Science. 1994;266:1202–8. 10.1126/science.7973702 7973702

[18] Henderson DW, Peacock S, Belton FC. Observations on the prophylaxis of experimental pulmonary anthrax in the monkey. J Hyg (Lond). 1956;54:28–36. 10.1017/S0022172400044272 13319688 PMC2217997

[19] Iacono-Connors LC, Novak J, Rossi C, Mangiafico J, Ksiazek T. Enzyme-linked immunosorbent assay using a recombinant baculovirus-expressed *Bacillus anthracis* protective antigen (PA): measurement of human anti-PA antibodies. Clin Diagn Lab Immunol. 1994;1:78–82. 7496927 10.1128/cdli.1.1.78-82.1994 PMC368200

[20] Harrison LH, Ezzell JW, Abshire TG, Kidd S, Kaufmann AF. Evaluation of serologic tests for diagnosis of anthrax after an outbreak of cutaneous anthrax in Paraguay. J Infect Dis. 1989;160:706–10. 2507648 10.1093/infdis/160.4.706

[21] Sirisanthana T, Navachareon N, Tharavichitkul P, Sirisanthana V, Brown AE. Outbreak of oral-oropharyngeal anthrax: an unusual manifestation of human infection with *Bacillus anthracis.* Am J Trop Med Hyg. 1984;33:144–50. 6696173 10.4269/ajtmh.1984.33.144

[22] Centers for Disease Control and Prevention. CDC interim recommendations for protecting workers from exposure to *Bacillus anthracis* in work sites where mail is handled or processed. Health Alert Network [online] Oct. 31, 2001 [cited 2002 May 17]; Available from: URL: http://www.cdc.gov/niosh/unp-mailrecs1.html 11708595

